# Promising therapeutic aspects in human genetic imprinting disorders

**DOI:** 10.1186/s13148-022-01369-6

**Published:** 2022-11-12

**Authors:** Yunqi Chao, Yifang Qin, Xinyi Zou, Xiangzhi Wang, Chenxi Hu, Fangling Xia, Chaochun Zou

**Affiliations:** 1 Department of Endocrinology, The Children’s Hospital, School of Medicine, Zhejiang University, Hangzhou, 310052 Zhejiang China; 2 Zhejiang University City College, Hangzhou, 310015 Zhejiang China

**Keywords:** Genetic imprinting disorders, Genomic imprinting, Therapy, Epigenetic therapy

## Abstract

Genomic imprinting is an epigenetic phenomenon of monoallelic gene expression pattern depending on parental origin. In humans, congenital imprinting disruptions resulting from genetic or epigenetic mechanisms can cause a group of diseases known as genetic imprinting disorders (IDs). Genetic IDs involve several distinct syndromes sharing homologies in terms of genetic etiologies and phenotypic features. However, the molecular pathogenesis of genetic IDs is complex and remains largely uncharacterized, resulting in a lack of effective therapeutic approaches for patients. In this review, we begin with an overview of the genomic and epigenomic molecular basis of human genetic IDs. Notably, we address ethical aspects as a priority of employing emerging techniques for therapeutic applications in human IDs. With a particular focus, we delineate the current field of emerging therapeutics for genetic IDs. We briefly summarize novel symptomatic drugs and highlight the key milestones of new techniques and therapeutic programs as they stand today which can offer highly promising disease-modifying interventions for genetic IDs accompanied by various challenges.

## Background

In mammals, the term genomic imprinting is an epigenetic phenomenon that, in some autosomal genes, gene expression depends on the parent-of-origin so that only one gene copy from the two parental alleles is preferentially active, either maternally or paternally [1]. In humans, there are approximately 150 imprinted genes residing on chromosomes 6, 7, 11, 14, 15, and 20 [2]. The realization has emerged that imprinted genes can function as pivotal regulators in prenatal and postnatal growth and development control, brain function, body composition, and energy homeostasis [3]. Disturbances in gene dosage, epigenetic regulation, and genomic sequences of imprinted genes may result in their function loss and can cause pathological conditions in humans—imprinting disorders (IDs).

Genetic IDs are a subset of congenital diseases caused by common molecular disturbances in genomic imprinting. Since the first human ID—Prader–Willi syndrome (PWS)—was identified in 1989 [4], there have been several other genetic IDs recognized according to genotype–phenotype studies: Angelman syndrome (AS), Beckwith–Wiedemann syndrome (BWS), Silver–Russell syndrome (SRS), pseudohypoparathyroidism types 1a (PHP1a) and 1b (PHP1b), transient neonatal diabetes mellitus (TNDM), Temple syndrome (TS14), Kagami–Ogata syndrome (KOS14), and Schaaf–Yang syndrome (SYS).

Currently, there is no dedicated and radical therapy for patients with genetic IDs, and all available first-line therapies per se are mainly supportive of the management and mitigation of partial existing symptoms. These symptomatic treatments usually cannot offer completely satisfactory symptom resolution for ID patients and have limited benefits to improve their quality of life. In the past decades, there has been a broad consensus on developing novel symptomatic drugs as candidate pharmacological approaches for genetic IDs. The pharmacotherapeutic armamentariums in progress, aiming at different pathophysiological pathways of every ID, hold great potential to optimize or renew present combinatorial therapies for treating certain symptom domains for ID patients.

Moreover, a great interest has been sparked regarding a subset of new and improved techniques and therapeutic programs that, as disease-modifying interventions, could render higher future potentials for treating genetic IDs. These therapies are developed based on the strategies of genetic precision medicine, which mainly target correcting or counteracting the defects caused by the function loss of associated imprinted genes: (1) gene replacement; (2) molecular reinstatement of the normal expression of candidate imprinted genes; (3) silencing the related inhibitory transcripts of imprinted genes, such as clustered regularly interspaced short palindromic repeats (CRISPR)–CRISPR-associated endonuclease (Cas) (CRISPR–Cas)-mediated gene editing; and (4) epigenetic circuit reprogramming. These therapies could represent relatively superior and more promising opportunities for managing genetic IDs. However, the majority of these novel therapeutic methods are still at the initial discovery level; therefore, further studies are needed to not only unveil detailed imprinting mechanisms from genetics and epigenetics backdrops but also perform more preclinical and clinical trials on these new interventions. In this review, we summarize recent findings on novel symptomatic drugs for IDs and highlight the advances in the fields of innovative promising therapeutic techniques and treatment programs in progress.

## The genomic and epigenomic basis of imprinting

Imprinted genes can be marked by different epigenetic machinery including DNA methylation, histone modifications, and chromatin structure. These modifications are set up during germline development and can be maintained as the memory of germline-derived parental-specific origin after fertilization, eluding genome-wide reprogramming [5]. Imprinted genes display the allelic parental expression pattern ubiquitously and permanently in nearly all cell types; however, some imprinted genes can exhibit imprinted expression patterns restricted to specific cell/tissue types [6, 7] or specific developmental windows [8].

**Fig. 1:**
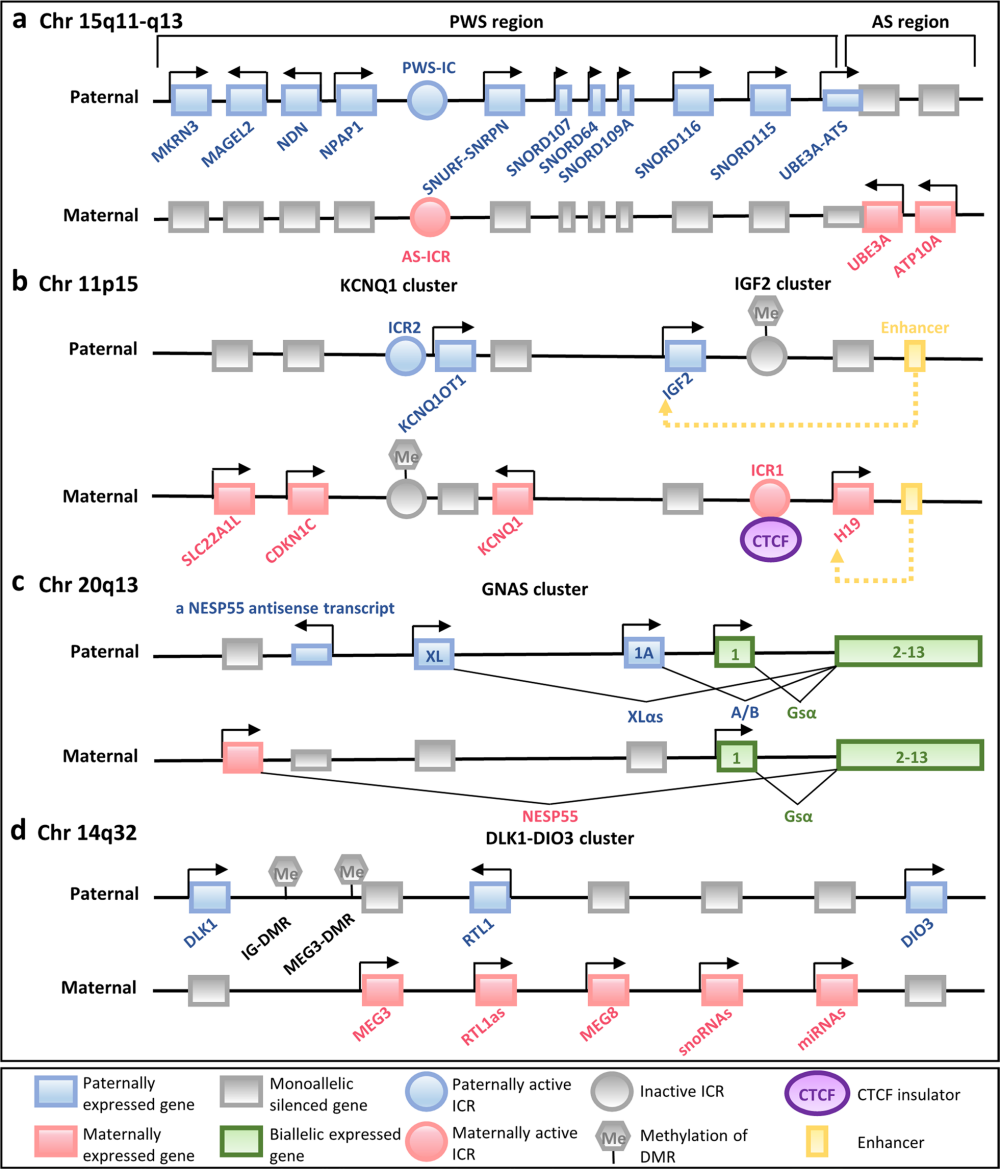
Schematic illuminations of representative imprinted gene clusters in humans. **a** The imprinted gene clusters within Prader–Willi syndrome (PWS)–Angelman syndrome (AS) (PWS–AS) region are shown. **b** The imprinted genes of potassium voltage-gated channel subfamily Q member 1 (KCNQ1) and H19 (which encodes an imprinted maternally expressed non-coding transcript)–insulin-like growth factor 2 (IGF2) (H19–IGF2) clusters, associated with Beckwith–Wiedemann syndrome (BWS) and Silver–Russell syndrome (SRS), are shown. **c** The GNAS (which encodes the G protein α-subunit Gsα) cluster of imprinted genes associated with pseudohypoparathyroidism is shown. Different transcripts originate from alternative 5’ exons display parental-specific expression pattern in certain tissues. **d** The delta-like 1 homologue (DLK1)–iodothyronine deiodinase 3 (DIO3) (DLK1–DIO3) cluster of imprinted genes associated with Temple syndrome (TS14) and Kagami–Ogata syndrome (KOS14) is shown. Chr, chromosome; ICR, imprinting control region; DMR, differentially methylated region, CTCF, CCCTC-binding factor; snoRNAs, small nucleolar RNAs; and miRNAs, microRNAs

Throughout the mammalian genome, the majority of imprinted genes are clustered together in imprinted domains, spanning 20–3,700 kb of DNA, and generally include several protein-coding genes and non-coding RNAs (ncRNAs) of different types (long non-coding RNAs (lncRNAs), microRNAs (miRNAs), and small nucleolar RNAs (snoRNAs)) [9, 10] (Fig. 1). The clustered imprinted genes in one imprinted domain are under the shared and coordinated regulation of an independent imprinting control region (ICR)—a germline differentially methylated region (DMR) [11]. Notably, four ICRs are paternally germline methylated, all within intergenic regions, and over twenty ICRs are of maternal germline origin, all comprising promoters of imprinted genes [12, 13]. The DNA methylation state of a cis-acting regulatory ICR determines the activity state of the imprinted genes within the entire domain—an ICR can actively direct monoallelic gene expression when unmethylated and become inactive when acquiring parent-of-origin-specific methylation.

The mechanisms by which unmethylated ICRs regulate imprinted gene expression are largely unknown. To date, two major mechanisms have been established: lncRNAs with silencing capacity and CCCTC-binding factor (CTCF)-dependent insulators. The lncRNAs, including KCNQ1 opposite strand 1 (KCNQ1OT1), UBE3A antisense transcript (UBE3A-ATS), gene-trap locus 2 (GTL2), and Airn, being imprinted themselves, can originate from the promoters within or near the ICRs. They have been shown to play an important role in silencing flanking imprinted genes in cis by different mechanisms: (1) The lncRNA product itself can directly suppress imprinted genes, such as UBE3A-ATS [14]; (2) the antisense transcriptional overlap of the lncRNA, rather than the lncRNA product, can confer transcriptional interference on the adjacent imprinted genes by disturbing RNA polymerase II recruitment, for example, the repressor function of Airn transcription in suppressing the transcription of the insulin-like growth factor type 2 receptor (Igf2r) gene[15]; and (3) lncRNAs can associate with local chromosome regions and recruit repressive histone modification machinery to the imprinted cluster, for example, GTL2 and KCNQ1OT1 which can form a discrete suppressive complex with Polycomb proteins [16, 17] to mediate the silencing of imprinted genes.

Moreover, CTCF, the zinc finger insulator protein, can participate in modulating the monoallelic expression of imprinted genes within the locus of H19 (which encodes an imprinted maternally expressed non-coding transcript)–insulin-like growth factor 2 (IGF2) (H19–IGF2) whose ICR harbors multiple CTCF binding sites [18] (Fig. 1b). CTCF can specifically bind to the unmethylated maternally inherited chromosome and form a higher-order chromatin structure, inducing a methylation-sensitive promoter–enhancer interaction: On the maternal chromosome, CTCF acts to prevent downstream enhancers from accessing IGF2 promoters and results in maternal IGF2 silencing, driving activity from H19 instead; in contrast, on the paternally inherited ICR, which is methylated, IGF2 promoters can be activated without CTCF binding.

## Types of molecular defects of genomic imprinting disturbances

**Fig. 2:**
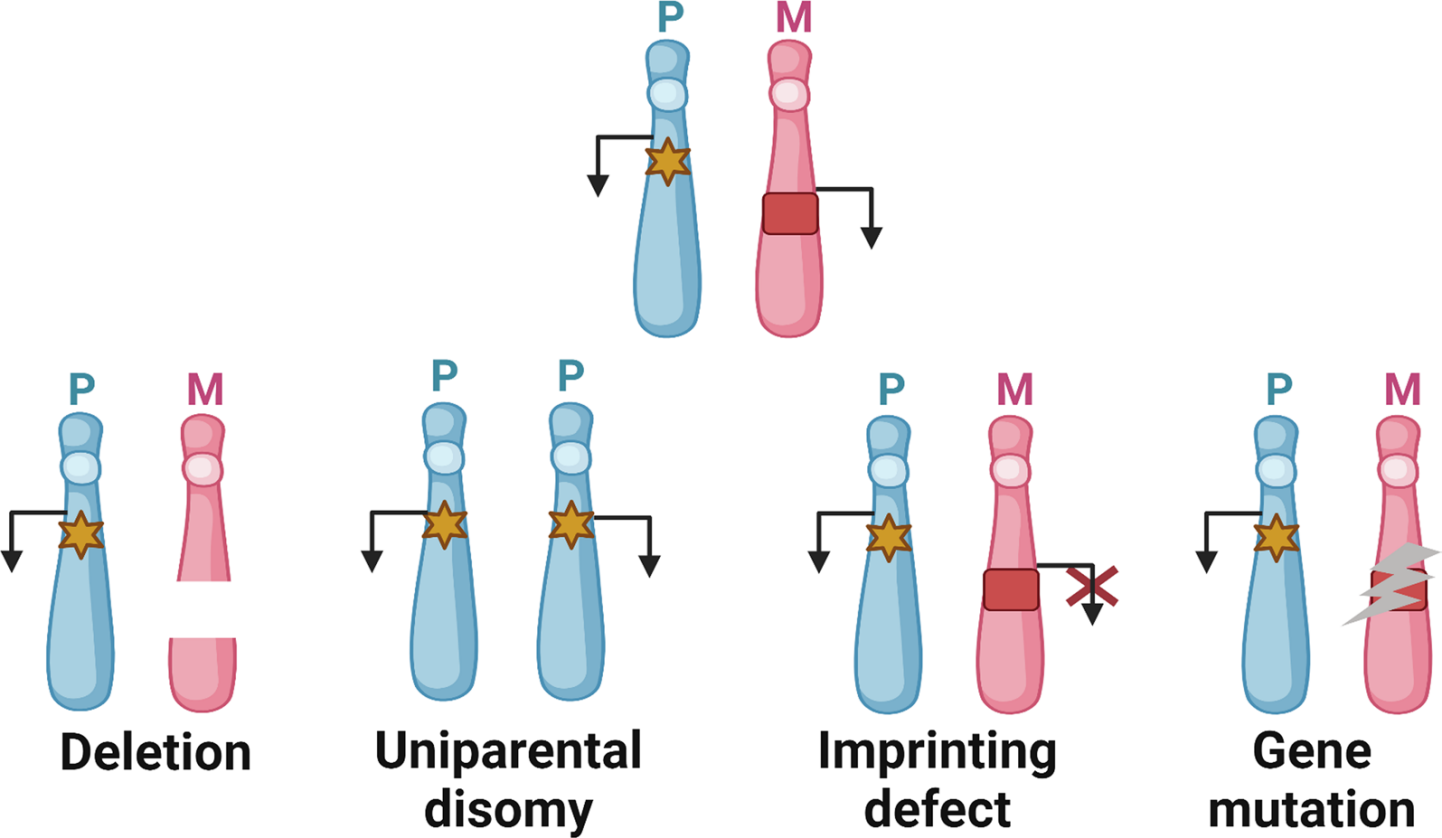
Exemplary schematic of four molecular subtypes in genetic imprinting disorders. The pathological conditions affecting the expression pattern of one maternal-inherited imprinted gene involve chromosomal deletion, paternal uniparental disomy (UPD) (where two paternal-inherited chromosomes are present losing the maternally expressed gene copy), imprinting defect (which is resulted from epigenetic dysregulation with identical DNA sequences), and gene mutation. For clarity, the paternal and maternal homologous chromosome ideograms (in blue and pink, respectively), one paternally expressed imprinted gene (yellow hexagram), and one maternally expressed imprinted gene (red square) are shown, respectively

Molecular defects underlying genetic IDs, causing unbalanced expression and functional disruption of imprinted genes, comprise four types of mutations and epimutations: (1) genomic mutations within imprinted genes, (2) chromosomal imbalances (deletions, duplications, and translocations), (3) uniparental disomy (UPD), and (4) epigenetic dysregulation of imprinted loci–imprinting defects (Fig. 2). The frequency of these molecular disturbances varies remarkably between different IDs. Moreover, it is noteworthy that somatic mosaicism can occur in individuals with IDs, in which cells with imprinting disruptions and cells with normal imprints are both contained in tissues. Mosaic distribution can account for somatic asymmetry and may obscure genotype–phenotype correlations.

### Imprinted gene mutation

IDs caused by pathogenic gene sequence variants of imprinted genes have been reported in BWS (inhibiting CDKN1C mutations [19]), SRS (activating CDKN1C mutation [20]), AS (UBE3A mutations [21]), PHP (inactivating GNAS (which encodes the G protein α-subunit Gsα) mutations [22]), and SYS (MAGEL2 mutation [23]). These intragenic mutations can occur de novo in specific germline or may be inherited from the father/mother who carries the disease-causing allele on the silent paternal chromosome. Indeed, the latter case shows an autosomal dominant inheritance mode with parent-of-origin-dependent penetrance of either maternal or paternal transmission and can account for familial reoccurring phenomena of IDs.

### Chromosomal imbalance

Chromosomal rearrangements producing copy number variants can cause the expression and complete function loss of imprinted genes by chromosomal deletions, translocations, or duplications. In some IDs, chromosomal deletion accounts for the majority of cases, for example, AS and PWS—with a maternal and paternal 15q11–q13 deletion, respectively. They can occur de novo or result from inherited chromosomal rearrangements.

### Uniparental disomy

UPD, mostly resulting from meiotic and mitotic nondisjunction errors, is a detectable genomic situation in which the inheritances of two both homologous chromosomes or chromosomal segments are derived from one parent. It can cause a double dose of imprinted genes and a complete function deficiency and has been reported in nearly all IDs associated with chromosomes 6, 7, 11, 14, 15, and 20 [24]. Specifically, for the fact that chromosomes 14 and 15 are predisposed to Robertsonian translocation (RobT) due to nondisjunction events, the RobT formation containing chromosome 14 and/or 15 is at increased risk of UPD, leading to related ID issues [25].

### Imprinting defects

An imprinting defect can cause incorrect activation of an allele-specific gene that should be silent or incorrect silencing of an allele-specific gene that should be active. Isolated or multi-locus epimutations can occur in gametes or often after fertilization, resulting in somatic mosaicism in ID carriers. The majority of epimutations can be involved in incorrect imprints of DMRs without evident alterations in genomic DNA sequence (primary imprinting defect). Moreover, epigenetic alterations can also indirectly result from genetic causes that may be traced back to mutations in cis- or trans-acting factors regulating the erasure, establishment, and maintenance of germline epigenetic imprints (secondary imprinting defect) [26]. In particular, a rare subset of imprinting disruptions occurring at multiple genome loci leads to multi-locus imprinting disturbances (MLIDs) in ID patients [27]. Notably, the involved loci can be linked with one known ID, for example, the BWS and SRS cases caused by the highest epimutation frequency in the ICR1 on chromosome 11p15.5 [28]. However, some epimutations may not have obvious correlations with phenotypes, possibly due to the specifically involved spectrum or their somatic mosaic nature.

## Environmental contributions to imprinting disturbances

In addition to genetic abnormalities, environmental factors can also cause imprinting disruptions. Numerous studies in humans have implicated an association between assisted reproductive technology (ART) and an increased risk of IDs, particularly BWS and AS [29–31]. ART manipulations, classically including in vitro fertilization (IVF) and intracytoplasmic sperm injection (ICSI), take place during the time window of epigenome reprogramming and can induce epigenetic variation in isolated hypomethylation on the KCNQ1OT1 and small nuclear ribonucleoprotein polypeptide N (SNRPN) genes [31, 32]. Other environmental predispositions, such as maternal nutritional status, paternal metabolic disorders, and prenatal exposure to endocrine disruptors, are reported to disturb imprinting marks affecting imprinted methylation patterns and may change the long-term epigenetic regulation of imprinted genes [33–35].

## Human genetic imprinting disorders

Genetic IDs usually affect multiple systems and produce spectrum disorders, often causing growth restriction during the fetal period and after birth, abnormal neuronal function and development, metabolic disorders, and in some cases, increased cancer susceptibility [36] (Table 1). The clinical phenotypes of these congenital conditions mainly depend on the affected parental allele, whereas different IDs can have overlapping phenotypes, for example, PWS, SRS, and TS14. Particularly noteworthy is that some of the IDs that result from the same dysregulated ICR are “mirror disorders,” for example, BWS and SRS, characterized by opposite gene expression patterns and clinical phenotypes.

**Table 1:** Major genetic imprinting disorders in humans

Imprinting disorder	Incidence	Clinical manifestations	Chromosomes and imprinted genes	Etiology
Prader–Willi syndrome (OMIM 176,270)	1/30,000–1/10,000	Neonatal hypotonia and poor suck, childhood-onset hyperphagia and severe obesity, developmental delay, hypogonadism, cognitive impairment, behavioral problems [37]	15q11–q13 (SNRPN, SNORD116)	Paternal deletion (65–70%), matUPD15 (20–30%), imprinting defect (1–5%)
Angelman syndrome (OMIM 105,830)	1/24,000–1/12,000	Developmental delay, severe intellectual disability, behavioral profile with happy demeanor, gait ataxia, microcephaly, seizure, characteristic electroencephalography [38]	15q11–q13 (UBE3A)	Maternal deletion (70–75%), gene mutation (5–10%), patUPD15 (1–2%), imprinting defects (1–3%)
Beckwith–Wiedemann syndrome (OMIM 130,650)	1/13,700–10,000	Pre/postnatal overgrowth, placental overgrowth, neonatal hypoglycemia, macroglossia, abdominal wall defects, predisposition to embryonal tumors [39]	11p15.5 (IGF2, CDKN1C)	Hypermethylation of H19/IGF2:IG-DMR causing biallelic expression of IGF2; hypomethylation of KCNQ1OT1:TSS-DMR causing CDKN1C silence, patUPD11, CDKN1C mutations
Silver–Russell syndrome (OMIM 180,860)	1/100,000–1/30,000	Severe IUGR, postnatal growth retardation, dysmorphism [40]	7p11.2 (MEST, GRB10)	MatUPD7 (5–10%), chromosomal rearrangements
			11p15.5 (IGF2, H19)	Paternal hypomethylation (35–65%), matdup11p15, gene mutations
Pseudohypoparathyroidism type 1a (OMIM 103,580)	Unknown	Resistance to parathyroid hormone (hypocalcemia and hyperphosphatemia), abnormal growth patterns, dysmorphism, obesity, cognitive impairment	20q13 (GNAS)	Maternal inactivating gene mutations of GNAS
Pseudohypoparathyroidism 1b (OMIM 603,233)		Resistance to parathyroid hormone (hypocalcemia and hyperphosphatemia)		Maternal deletions, methylation defects within the GNAS complex locus, patUPD20
Transient neonatal diabetes mellitus (OMIM 601,410)	1/300,000	Neonatal hyperglycemia and IUGR	6q24 (PLAGL1, HYMAI)	PatUPD6 (40%), paternal duplication (32%), methylation defects (28%)
Temple syndrome (maternal UPD 14 syndrome) (OMIM 616,222)	Unknown	Prenatal and postnatal growth retardation, characteristic facies, premature puberty, obesity [41]	14q32 (DLK1, RTL1/GTL2)	MatUPD14, paternal deletion, aberrant methylation,
Kagami–Ogata syndrome (paternal UPD 14 syndrome) (OMIM 608,149)	Unknown	Polyhydramnios, placentomegaly, mental retardation, abdominal wall defects, dysmorphism [42]		PatUPD14, maternal deletion, aberrant methylation
Schaaf–Yang syndrome (OMIM 615,547)	Unknown	Developmental delay, intellectual disability, hypotonia, feeding difficulties, autism spectrum disorder [23]	15q11.2 (MAGEL2)	Paternal truncating gene mutations (100%)

OMIM, Online Mendelian Inheritance in Man; SNRPN, small nuclear ribonucleoprotein polypeptide N; SNORD116, small nucleolar RNA, *C*/*D* box 116 cluster; mat, maternal; UPD, uniparental disomy; UBE3A, ubiquitin protein ligase E3A; pat, paternal; IGF2, insulin-like growth factor 2; CDKN1C, cyclin-dependent kinase inhibitor 1C; H19 encodes an imprinted maternally expressed non-coding transcript; IG-DMR, intergenic differentially methylated region; KCNQ1OT1, KCNQ1 opposite strand 1; TSS-DMR, transcription start site differentially methylated region; IUGR, intrauterine growth restriction; MEST, mesoderm-specific transcript; GRB10, growth factor receptor-bound protein 10; PTH, parathyroid hormone; GNAS encodes the G protein α-subunit Gsα; PLAGL1, pleomorphic adenoma gene‑like 1; HYMAI, hydatidiform mole-associated and imprinted; DMRs, differentially methylated regions; DLK1, delta-like 1 homologue; RTL1, retrotransposon-like 1; GTL2, gene-trap locus 2; and MAGEL2, MAGE family member L2.

The therapeutic strategies for ID patients are substantially different between the conditions in which unbalanced imprinted gene expression modes appear before lineage commitment (paternally/maternally inherited or occurring de novo before fertilization) and the somatic mosaicism conditions occurring after fertilization (resulting from epimutations and/or UPDs). Effective and complex coordination of health care targeting multisystem manifestations constitutes a common consensus for the former condition. Regarding ID mosaicism conditions, which can be seen in BWS and SRS, limb or body asymmetry can often present in these cases as an isolated finding, and treatment choices for these patients should determine whether the asymmetry condition represents decreased growth (hemihypoplasia) or overgrowth (hemihyperplasia) [39]. Surgical corrections are usually planned for subjects with lateralized overgrowth; tumor management is required for BWS children with any kind of tumor, mainly Wilms tumor, hepatoblastoma, and adrenal carcinoma. Indeed, these therapeutic strategies are all symptomatic and cannot prevent the high recurrence risk of related pathological conditions.

## Emerging pharmacotherapies for genetic imprinting disorders

Over the decades, the lack of known molecular targets related to genetic IDs has hindered the development of specific pharmacotherapies for patients. However, a recent surge of treatments with novel symptomatic drugs that traditionally have curative effects in other disorders has begun to suggest their therapeutic potential for patients with genetic IDs because the molecular modes of action of these new therapeutic agents can have correlations with those in genetic IDs (Tables 2, 3, 4). These emerging drug candidates, despite not being disease-modifying, are developing at different research phases and hold promise to counter molecular defects in genetic IDs and afford clear therapy benefits for clinically treating certain phenotypes in patients.

**Table 2:** Novel symptomatic pharmaceuticals developed in clinical trials for PWS

Modality	Clinical trial no	Phase	Potential curative effects
Oxytocin	NCT02205034;	Phase 1, 2	Improve suckling in PWS infants; reduce appetite drive, improve social skills, and decrease disruptive behaviors in PWS children [43, 44]
	NCT02013258	Phase 1	
	NCT02804373	Phase 2, 3	
Oxytocin analogue (Carbetocin)	NCT03649477	Phase 3	Improve hyperphagia and behavioral symptoms [45]
K^+^-ATP channel agonists (Diazoxide, DCCR)	NCT03440814	Phase 3	Ameliorate hyperphagia, improve lipids and insulin resistance, reduce aggressive behaviors [46]
	NCT02034071	Phase 1, 2	
	NCT03714373	Phase 3	
UAG analogue (AZP-531)	NCT03790865	Phase 2,3	Improve hyperphagia and metabolic parameters [47]
GLP-1 receptor agonists (Liraglutide, Exenatide)	NCT02527200	Phase 3	Improve hyperphagia [48]
	NCT01444898	Not applicable	
	NCT00551343	Not applicable	
MetAP2 inhibitor (ZGN-440)	NCT01818921	Phase 2	Reduce body weight and improve hyperphagia-related behaviors [49]
MC4R agonist (Setmelanotide)	NCT02311673	Phase 2	Improve hyperphagia and result in weight loss [50, 51]
GOAT inhibitors (GLWL 01)	NCT03274856	Phase 2	Reduce food intake [52]
CB1R antagonists (JD5037, CBD)	NCT02844933	Phase 2	Suppresses appetite, improve metabolic issues, increase energy expenditure [53, 54]
	NCT03458416	Phase 2	
	NCT05098509	Phase 2,3	
Antiepileptics (Topiramate)	NCT02810483	Phase 3	Attenuates self-injurious behaviors, correct eating behaviors, control body weight [55, 56]
	NCT00065923	Not applicable	

DCCR, diazoxide choline controlled release; UAG, unacylated ghrelin; GLP-1, glucagon-like peptide 1; CFE, Caralluma fimbriata extract; MetAP2, methionine aminopeptidase 2; MC4R, melanocortin-4 receptor; GOAT, ghrelin O-acyltransferase; CB1R, cannabinoid type1 receptor; and CBD, cannabidiol

**Table 3:** Novel symptomatic pharmacological treatments for AS

Modality	Research stage	Potential curative effects
Gaboxadol (OV101)	Clinical trial (NCT03882918, NCT02996305: phase 2; NCT04106557: phase 3)	Improve motor functions and rescue behavioral and sleep deficits [57]
Modified diketopiperazine (NNZ-2591)	Clinical trial (NCT05011851: phase 2)	Improve motor and cognitive deficits and decrease seizures
Nutritional formulation of exogenous ketones	Clinical trial (NCT03644693: Not applicable)	Improve motor coordination, learning skills and overall neurologic functions and reduce seizure activity [58, 59]
Melatonin	Clinical trial (NCT01903681: phase 1; NCT01906866: phase 3)	Ameliorate nighttime sleep disorders [60, 61]
Levodopa/Carbidopa	Clinical trial (NCT00829439: phase 1; NCT03235037: Not applicable; NCT01281475: phase 2,3)	Improve neurodevelopment and reduce abnormal movements (e.g., tremors) [62]
Minocycline	Clinical trial (NCT02056665: phase 2; NCT01531582: Not applicable)	Decrease motor deficits and increase long-term potentiation [63]
NSI-189	Preclinical	Improve motor and cognitive functions [64]
CB1R antagonist (CBD)	Preclinical	Attenuates seizures and help normalize the EEG deficits [65]
CIM6P/IGF2 receptor ligands	Preclinical	Reserve cognitive impairment, motor deficits and attenuate audiogenic seizures [66]
BK-channel antagonist	Preclinical	Help normalize neuronal excitability and ameliorate seizure susceptibility [67]
Lovastatin and simvastatin	Preclinical	Suppress the epileptiform activity and audiogenic seizure [68]; improve the cognitive and behavioral deficits [69]
PP2A inhibitor (LB-100)	Preclinical	Improve motor functions and rescue behavioral deficits [70]
Taurine	Preclinical	Improve learning and motor skills [71]

NNZ-2591, cyclo-L-glycyl-L-2-allylproline; NSI-189, NSI-189 phosphate; CB1R, cannabinoid type1 receptor; CBD, cannabidiol; EEG, electroencephalography; CIM6P, cation-independent mannose-6-phosphate; IGF2, insulin-like growth factor 2; BK, calcium- and voltage-dependent big potassium; and PP2A, protein phosphatase 2A

**Table 4:** Novel symptomatic pharmacological treatments for other genetic IDs

Syndrome	Modality	Research stage	Putative curative effects
BWS	mTOR inhibitor (sirolimus)	Preclinical	Stabilize the blood glucose concentrations [72]
SRS	Aromatase inhibitor (Anastrozole)	Clinical trial (NCT01520467: not applicable)	Limit the progression of bone maturation
	First-generation antihistamine (CYP)	Pilot study	Increase growth velocity and improve nutritional status [73]
PHP	PDE inhibitor (Theophylline)	Clinical trial (NCT03029429, NCT02463409: phase 2; NCT03718403: phase 4)	Control early-onset obesity, decrease hormone resistance, slow the rate of epiphyseal closure
	Calcimimetic agent (Cinacalcet)	Pilot study	Help to control serum PTH level [74]
TNDM	SU	Pilot studies	Help to normalize insulin secretion and control glycemia [75–78]
	DPP4 inhibitor (Alogliptin)		
SYS	Oxytocin	Preclinical	Improve the development of the nervous system [79]

CYP, cyproheptadine; PDE, phosphodiesterase; PTH, parathyroid hormone; SU, sulfonylurea; DPP4, dipeptidyl peptidase-4

Indeed, for PWS patients, several pharmacological treatments in the pipeline can exert significant effects on specific symptom domains, for example, life-threatening hyperphagia and morbid obesity, aberrant body composition, and cognitive and behavioral abnormalities. In addition, for AS patients, the present exploration of pharmaceuticals is a great attempt to manage seizures with the goal of reducing the side effects of current antiepileptic drugs application. New drugs have also been developed to improve the therapeutic outcomes of ameliorating cognitive impairment, sleep disturbance, and motor deficits in AS patients. Regarding other genetic IDs, novel symptomatic drugs could also facilitate the rescue of functional phenotypes to some degree in a few affected domains. These novel symptomatic drugs indeed have the potential to improve patients’ quality of life and be further used as major therapy methods or, more likely, combined therapeutic interventions for treating patients.

**Fig. 3:**
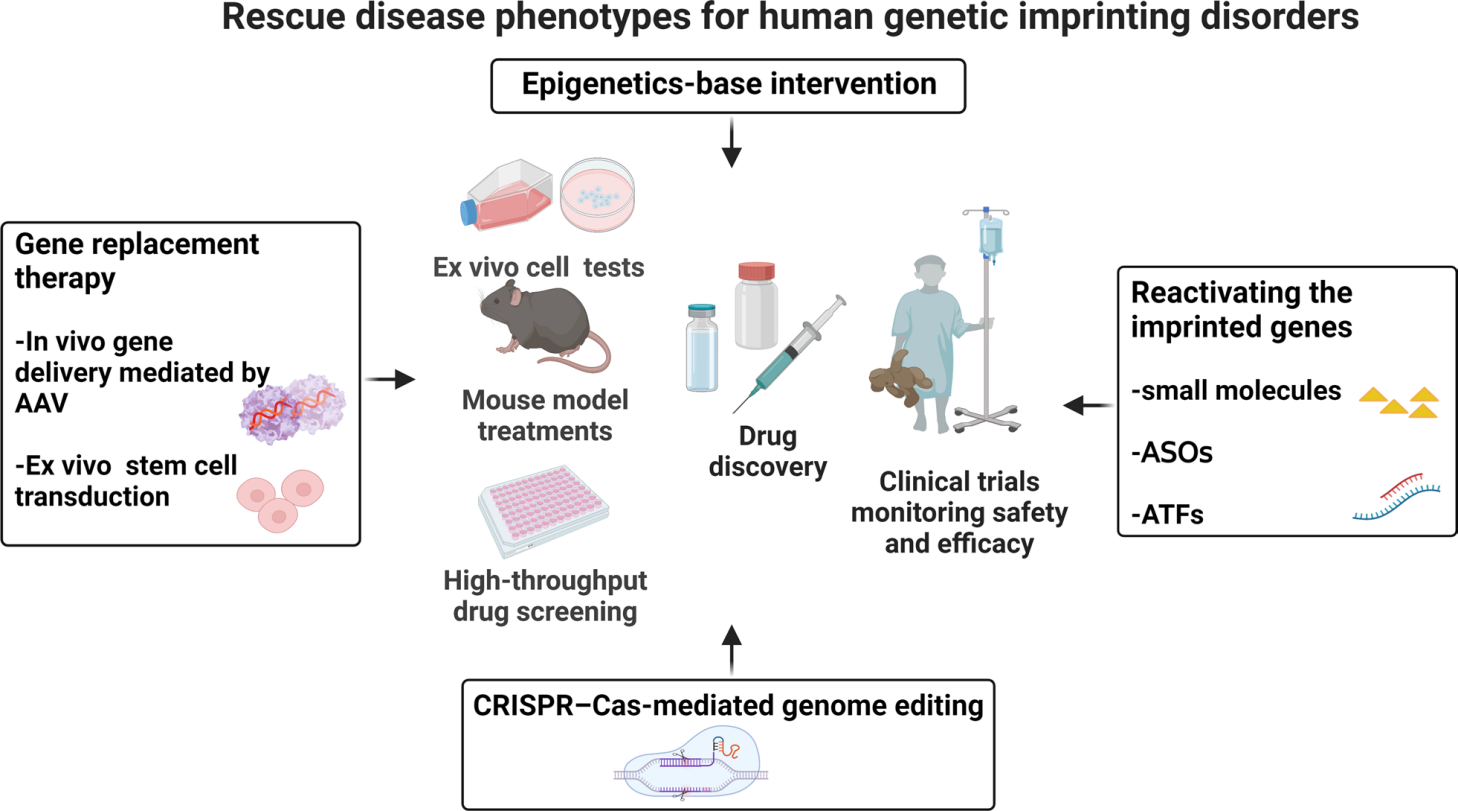
Novel therapeutic strategies for treating human genetic imprinting disorders. AAV, adeno-associated virus; ASOs, antisense oligonucleotides; ATFs, artificial transcription factors; CRISPR–Cas, clustered regularly interspaced short palindromic repeats (CRISPR)–CRISPR-associated protein (Cas)

Moreover, a subset of precision therapy approaches employing highly appealing therapeutic techniques has come into view and renewed our understanding of treating genetic IDs (Fig. 3). Indeed, they could represent relatively valid and superior approaches beyond the reach of traditional therapy options for the management of genetic IDs.

## Ethical considerations and concerns regarding novel ID therapeutic strategies

Like any new therapeutics for human diseases, ethical aspects warrant great concern before ensuring responsible use of the emerging techniques to treat and cure IDs in human patients. Several essential but controversial ethical issues need careful consideration.

First, the broad consensus is that studies on these novel therapeutic strategies for IDs should primarily provide scientific evidence on potential health benefits with compelling medical needs. This necessitates considering whether a partial or complete phenotypic correction is of considerable curative significance for ID patients. Second, rigorous evaluations must be carried out on the issues of safety, tolerability, feasibility, and efficacy [80]. In this regard, some forms of therapeutic technologies leading to higher risks of genetic modifications of the human genome, such as the CRISPR–Cas9-mediated genome-editing approach, cannot meet basic safety and efficacy standards and have proven to be ethically unacceptable thus far, as reviewed and discussed by an international committee cosponsored by the US National Academy of Sciences and the National Academy of Medicine [81]. Moreover, phenotypic toxicity and undesired “off-target” effects are recurrent questions producing potential risks and adverse effects in many novel therapeutic choices for IDs, such as virus-mediated gene replacement therapy, pharmacological small-molecule approaches, and epi-drugs, and the prevention of these risks and adverse effects must be emphasized in preclinical studies. Third, choosing the appropriate treatment window can be an important factor in achieving satisfactory effects, especially for ID patients with disturbed growth and development. Basically, restorative interventions for IDs should be administered early because missing a critical treatment period may lead to less-than-expected therapeutic effects. Lastly, further establishment of exact standards for future clinical applications under credible and concomitant ongoing surveillance and administration is urgently necessary. Indeed, there is a long way to go for setting corresponding bioethical guidelines and regulatory policies to protect ID patients, which should be prepared for advanced therapeutics before translating into clinical practice.

## Gene replacement therapy

Gene therapy, defined as introducing functional genetic materials into the target cells of patients as a therapeutic pathway, is considered a highly promising treatment for many genetic diseases and also some previously untreatable diseases [82, 83]. These findings provide an excellent proof-of-concept of gene replacement therapy for treating genetic IDs. In some ID cases resulting from the mutation and deficiency of imprinted genes, the therapeutic strategy of sole reinstatement of the expression of the candidate gene might ameliorate or cure disease-related phenotypes. The two basic delivery strategies for therapeutic genes are adeno-associated virus (AAV)-based in vivo gene delivery and ex vivo stem cell transduction pathway.

### Adeno-associated virus-mediated gene replacement strategy

As a gene delivery vector, AAV can transfer genes through recognizing and infecting cells efficiently and has been increasingly successful with the properties of long-term efficacy, low-level pathogenicity, and a strong safety profile, making it suitable for therapeutic application. Considerable clinical potential has been established for the treatment of Leber congenital amaurosis (LCA) [84], hemophilia B [85], cancers [86, 87], etc.

Regarding AAV-based gene therapy for genetic IDs, AAV vectors can be engineered to carry the normal gene and replace the pathogenic mutated one in patients’ cells. Taking AS cases as an example, the majority of which lack maternal UBE3A, an early attempt utilized an AAV vector carrying an exogenous UBE3A copy injected into the hippocampus of adult AS mice to restore local UBE3A expression [88]. The therapeutic consequences involve enhanced hippocampus-dependent associative learning and memory ability, but motor deficits show slight improvement due to the limited distribution beyond the hippocampus [88]. In another optimized study, a dual-isoform of human UBE3A (hUBE3A) enabling the translation of both short and long hUBE3A isoforms was packaged into a recombinant AAV9-derived PHP.B vector and was under intracerebroventricular administration into the developing mouse brain [89]. Upon widespread UBE3A re-expression in the brain, more significant therapeutic outcomes were supported, especially improvements in motor learning and behavioral performance [89].

Notably, a reliable AAV-mediated gene replacement strategy should provide two fundamental therapeutic aspects: efficacious relief of symptoms and tolerance to transgene overexpression in vivo. Thus, before being translated into clinical benefits for patients with genetic IDs, more attempts focusing on engineering AAV vectors and devising therapeutic transgenes with better bioavailability potential should be further explored.

### Stem cell transduction

Stem cell gene therapy is a promising therapeutic strategy for genetic disorders. It exploits hematopoietic stem and progenitor cells (HSPCs) engineered and modified ex vivo to transfer a healthy gene copy by integrating a viral vector–like lentiviral vector (LV) or to function as cell vehicles to deliver therapeutic molecules into the targeted cells or tissues [90]. Particularly noteworthy is that autologous transplanted HSPCs can differentiate into physiological cell lines such as immune cells, making it possible for them to cross the blood–brain barrier (BBB) to reach the central nervous system (CNS), thus offering wider application in the treatment of CNS diseases. Moreover, this gene therapy pathway involving autologous transplantation can reduce the risks of immune reactions; at the same time, due to the genome integration ability of LVs, it also provides a theoretically permanent treatment for patients. Many treatments of gene-corrected HSPCs have shown favorable safety profiles and excellent therapy efficacy in clinical use for various diseases, including primary immune deficiencies [91], erythrocyte disorders [92], and inherited metabolic diseases [93].

Recently, one study utilized this gene therapy approach for treating AS by transplanting genetically modified HSPCs transduced with a functional UBE3A LV into an immunodeficient AS mouse model [94]. Following transplantation, UBE3A-expressing microglia could be derived from CD34^+^ HSPCs and further provided UBE3A protein at therapeutic levels to the affected neurons in the brain. A significant enhancement of cognitive behaviors and motor functions was observed in both neonatal and adult mice. This autologous stem cell-based gene therapy suggests a broadened promising treatment to achieve life-long UBE3A delivery in the CNS for AS patients, independent of a critical treatment window.

## Therapeutic methods of reactivating imprinted genes

Because inhibitory lncRNAs within imprinted domains are responsible for silencing flanking imprinted genes in cis, the reinstatement of candidate imprinted genes through targeting these lncRNAs conceptually harbors certain feasibility and utility. This raises the possibility that suppressing the expression of lncRNAs with silencing capacity can be realized and become a potential therapy for ID patients. Some exploratory attempts have been made to validate this treatment strategy via various methods, such as high-content small-molecule screening, antisense oligonucleotides (ASOs), and artificial transcription factors (ATFs).

### Pharmacological small-molecule approaches

Small molecules, with the advantages of favorable chemical stability and bioavailability, can effectively regulate different signaling pathways [95]. Through large-scale, high-content screening, certain modulatory compounds can be discovered and selected from a chemical library of small molecules. The past decades have witnessed significant advances in small-molecule drugs as molecular tools for deciphering mechanisms of human diseases and normal biology, and as new therapeutics for treating cancers [96, 97] and monogenic diseases [98] and addressing drug resistance (as in infectious diseases [99] and cancers [100]). Hence, the therapeutic potential of pharmacological small molecules for treating IDs is notable.

For AS patients, due to tissue-specific genomic imprinting, the maternal expression of the UBE3A gene is absent in neurons, while the equivalent paternal copy is intact but repressed by the transcription of the lncRNA UBE3A-ATS in the antisense direction [14]. Through an unbiased, high-content screen, topoisomerase inhibitors, such as topotecan, a chemotherapeutic anticancer drug, have been selected and identified to inhibit Ube3a-ATS transcription and increase paternal expression of catalytically active Ube3a in the cortical neurons of mice [101]. In addition, transient topotecan treatment also showed enduring effects of restoring the paternal Ube3a allele in vivo. However, topoisomerase inhibitors exhibit toxicity and lack specificity in that they can concomitantly affect the transcription of many other long genes across the genome in addition to Ube3a-ATS [102], raising the concern of “off-target” effects and suggesting the limitations of their further advancement in AS treatment.

### Antisense oligonucleotide (ASO)

ASOs are chemically modified short single-stranded nucleic acids that can specifically suppress the expression of certain genes or modulate splice switching [103]. ASOs can bind to targeted RNA through Watson–Crick base-pairing and cause RNA degradation by recruiting RNase H endonuclease. Emerging as a novel class of drugs, antisense drugs have been widely investigated for their curative effects in many human diseases, several of which have already been implemented in the clinic to treat patients with hereditary transthyretin-mediated (hATTR) amyloidosis, Duchenne muscular dystrophy (DMD), and spinal muscular atrophy (SMA) [104]. Moreover, ASO drugs can be delivered to the target system via many methods and especially exhibit therapeutic advantages in neurological diseases following central administration [105, 106].

ASOs can also be considered suitable candidates for AS treatment strategies. It has been demonstrated that ASO treatment is generally well tolerated and can effectively achieve specific inhibition of Ube3a-ats to sustain the expression of paternal Ube3a, both at the molecular level in neurons and in brains in vivo [107, 108]. Importantly, the optimal ASO treatment for reactivating Ube3a expression in murine AS models requires a critical developmental window of the earlier neurodevelopmental phase around birth [109, 110]. Through intracerebroventricular injection in neonatal AS mice, brain-wide Ube3a distribution can be achieved and AS-related neurocognitive deficits, and epilepsy can also be partially but persistently ameliorated [108]. Therefore, the ASO approach could represent a promising disease-modifying treatment to rescue most disease-associated phenotypes for patients with AS.

To date, three ASO compounds for AS treatment have entered phase 1 clinical trials (GTX-102, NCT04259281; RO7248824, NCT04428281; and ION582, NCT05127226). However, we should also note that the direct translation of ASO treatment for ID patients is still limited. One crucial problem remains for preclinical studies: The precise alignment of brain development between mice and humans is largely unknown; therefore, the phenotypic assessment among mice, especially behavioral tests, cannot be applied to humans with adequate clinical value [110]. Additionally, when proceeding into clinical tests, another concern is about the most efficient mode and route of the ASO delivery system for humans, combined with its dose, duration, and distribution throughout targeted tissues and systems in vivo. Moreover, as ASOs have short half-lives, they need to be administered on a regular schedule to achieve long-term effects on gene regulation in patients. Indeed, before translating into a clinically feasible treatment for IDs, more detailed information is necessary regarding the issues of ASO management regimens and evaluations of efficacy, safety, and tolerability.

### Artificial transcription factors (ATFs)

An ATF is a genome interrogation-mediated tool to modulate gene expression in trans. It can be designed as a binary system composed of a DNA-binding domain (DBD), such as zinc fingers, transcription activator-like effectors (TALEs) or CRISPR–Cas systems, and an effector region that can decide the up- or downregulatory transcription of endogenous target genes [111]. When the DBD is linked to an activating domain, the ATF becomes a transcriptional activator to increase targeted gene expression, whereas the linkage of a repressive domain makes a transcriptional repressor to downregulate targeted gene expression. Moreover, the effector domain can also exhibit enzymatic activities involved in epigenetic editing and chromatin remodeling [112].

In one study, an injectable ATF was established, functioning in suppressing Ube3a-ATS production to restore Ube3a expression, which supports a therapeutic evidence line for AS [113]. Structurally, this factor was engineered to involve a zinc finger domain to bind to the promoter region of small nuclear ribonucleoprotein polypeptide N (SNRPN). Through subcutaneous or intraperitoneal injection in adult AS mice, the ATF could cross the BBB and distribute diffusely throughout the hippocampus and cerebellum [103]. Although utilizing ATF biologics can show short-term rescue effects in the brain from molecular aspects, the phenotypic investigations on behavior and cognition remain unassessed and unknown in treated mice.

## CRISPR–Cas9-mediated genome-editing approach

The genome-editing system of RNA-guided CRISPR–Cas, functionally introducing DNA double-strand breaks (DSBs) and stimulating non-homologous end joining (NHEJ) and homologous-directed repair (HDR), can correct gene mutations and modulate gene expression in eukaryotic cells [114]. Representing genetic precision medicine, CRISPR–Cas9 technology holds great promise to offer permanent reversal of pathologic DNA mutations for genetic disorders. In particular, the CNS has been recognized as a potential target for therapeutic genome-editing methods following focal delivery of CRISPR–Cas9 into different brain regions. Accumulating lines of therapeutic evidence of gene editing in the CNS have been reported in mouse models of various diseases, such as amyotrophic lateral sclerosis [115], Huntington’s disease [116], and familial Alzheimer’s disease [117].

As recently demonstrated, an established CRISPR–Cas9 system targeting the UBE3A-ATS transcript can block its expression and reactivate paternally inherited UBE3A expression in primary human neurons and in an AS mouse model [118, 119]. In addition, this efficacious gene-editing tool can be delivered via AAV into the mouse brain during early postnatal stages, and due to the functional restoration of paternal UBE3A throughout the nervous system, long-lasting therapeutic benefits in anatomical and behavioral deficits of AS can be achieved [118].

Although CRISPR–Cas9-mediated gene-editing technology has been implicated to have optimistic therapeutic benefits in genetic IDs, no gene-specific therapy exists for ID patients. Considering the genome and phenotype differences between humans and mice, numerous obstacles limiting the therapeutic outcome of this approach still lie ahead, the main aspects of which involve (1) design and screening of appropriate single-guide RNAs (sgRNAs) for human mutant alleles; (2) efficient and safe transport and delivery pathways of the editing system; (3) genome-editing efficiency; (4) unintended off-target activity; (5) therapeutic threshold of editing; (6) toxic reactions; and (7) other unknown potential risks. Beyond any doubt, surmounting these pitfalls is an essential prerequisite for future translational studies.

## Epigenetics-based intervention

Within the past decades, reprogramming the epigenetic landscape to modulate the disease-causing defects present in the genome has revolutionized the research field of developing innovative treatments for different diseases. The so-called epi-drugs can function as activators or inhibitors of epigenetic regulatory proteins to manipulate aberrant DNA methylation and histone modifications. Several epi-drugs, such as DNA methylation inhibitors and histone deacetylase inhibitors, have been approved for clinical applications to treat solid tumors and hematologic cancers and have afforded clear therapeutic benefits in patients [120–122]. Moreover, numerous investigations of potential epigenetic therapy in other pathologies, involving infectious diseases [123], cardiovascular disease [124], metabolic disorders [125], and neurodevelopmental and neuropsychiatric diseases [126, 127], either have received approval or are being tested in clinical trials.

Given that individuals with genetic IDs retain at least one intact but epigenetically silent gene copy, reversing their imprinting status to compensate for the deficiency from the affected parental copy is a potential opportunity for curing and treating IDs. Hence, reprogramming the related epigenetic circuits to induce the reinstatement of inactivated candidate genes can become a research hotspot.

### Epigenetic therapy for PWS

Within the PWS region on chromosome 15q11–q13, there are 15 known disease-related genes, among which the small nucleolar C/D box RNA 116 (SNORD116) cluster may be the paramount causative player in PWS [128]. Unveiling related epigenetic mechanisms and reactivating functionally dormant genes on maternal alleles can offer potential therapeutic targets. At present, several groups have proposed new methods of epigenetics-based intervention for the treatment of PWS.

An epigenetic complex comprised of zinc finger protein 274 (ZNF274) and SET domain bifurcated 1 (SETDB1)—a histone H3 lysine 9 (H3K9) methyltransferase, has been demonstrated to be a separate imprinting mark to induce the expression silence of maternal PWS genes [129]. Studies knocking down SETDB1 [129] or knocking out ZNF274 [130] in neurons from PWS induced pluripotent stem cells (iPSCs) found that H3K9 tri-methylation (H3K9me3), a featured histone mark of closed chromatin, at the SNORD116 locus decreases and maternal SNORD116 expression can be partially restored. In contrast to SETDB1-deficiency, ZNF274 inactivation can acquire specificity without affecting DNA methylation at the PWS-ICR [129, 130].

Additionally, through a high-content screen, two selective small-molecule inhibitors of euchromatic histone-lysine N-methyltransferase-2 (EHMT2/G9a), UNC0642 and UNC0638, have been discovered to reactivate maternal PWS imprinted genes both in a PWS mouse model and in PWS-derived iPSCs [131]. The two G9a inhibitors can selectively reduce H3K9 di-methylation (H3K9me2) at the PWS-ICR locus without affecting DNA methylation [131]. Moreover, therapeutic effects have been indicated in PWS newborn mice managed by G9a inhibitors, and survival and growth have been significantly improved [131]. This result provides a functionally optimistic basis for developing small-molecule-based epigenetic therapy for PWS patients, and further investigation of this approach is warranted.

Moreover, versatile CRISPR–Cas9 technology has emerged as a powerful leading platform for engineering the epigenome with the advent of catalytically dead Cas9 (dCas9) (without DNA cleavage function). Via fusing to various epigenetic effectors, such as DNA demethylases/methyltransferases and histone-modifying enzymes [132], the CRISPR–dCas9 system can target the specific promoter/exon 1 sequences to modulate gene expression sterically. This site-specific epigenome-editing tool could broaden the targeted therapeutic space in human diseases because several proof-of-principle studies in vivo have provided its therapeutic effects in the management of chronic pain [133] and neurological disorders [134, 135]. Indeed, as an alternative to pharmacological small-molecule approaches, the utilization of CRISPR–dCas9 in epigenetic reprogramming also provides novel therapeutic directions for genetic IDs based on the strategies of reactivating the imprinted genes from the other parent-of-origin chromosome or suppressing the improper biallelically expressed genes. There was a study fusing dCas9 with an epigenetic effector–DNA plus dioxygenase 1 (TET1) (dCas9–TET1) to effectively edit the targeted epigenetic marks of the imprinted PWS region, allowing the activation of the SNRPN gene in vivo [136].

### Future prospects in epigenetic treatments for other genetic imprinting disorders

From the view of treating genetic IDs, advances in understanding disease-related imprinting mechanisms and the deregulation of epigenetic processes will provide an opportunity for using an epigenetic approach to functionally induce the restoration of silenced genes. Therefore, by targeting these syndromes at their “roots” to reinstate the normal transcriptional activity of imprinted genes, epigenetics-based intervention toward new drug discovery could arguably be a game-changer for the therapeutic strategy of IDs.

Ideally, for IDs patients harboring only one intact but inactive candidate gene, using epigenetic modulating agents against the activity of epigenetic modifiers or remodelers may offer a valid opportunity to restore the silenced copy, for instance, IGF2 re-expression for SRS patients, GNAS re-expression for PHP patients, and UBE3A re-expression for AS patients. In detail, for AS cases with maternal UBE3A deletion, sgRNA-guided dCas9 may tether with epigenetic modifiers or regulators, such as DNA methyltransferase writers, to target the CpG islands at the UBE3A-ATS promoter. This could theoretically result in increased UBE3A expression through mimicking the maternal silencing methylation pattern. As another case in point, for SRS patients caused by a loss of methylation (LOM) on paternal H19–IGF2 DMR in chromosome 11p15, epi-editors of DNA methyltransferase may function to reverse the imprinted status of the IGF2 gene. Besides, in some pathological situations in which interstitial duplications cause imprinted gene overexpression, complete methylation-induced silencing of candidate genes may not be favorable; rather, a decrease in expression to ordinary levels should be considered the main goal of epigenetic therapy.

### Challenges in epi-drugs development

The development of epi-based applications for genetic IDs may face several formidable challenges.

The first challenge stems from the scientific aspect—the complexity of epigenetic regulatory processes and the heterogeneity of genome and epigenome landscapes, which remain elusive for most genetic IDs. Actually, these open questions are fundamental and pivotal for determining the right and most beneficial genomic and epigenomic programs needing regeneration in ID patients.

Second, as a rescue option, efficacy evaluation is also a critical issue: whether epigenetic manipulation could largely or completely restore imprinted gene expression to sufficient levels to render global effects on phenotypic correction or whether merely partial reinstatement is enough to mitigate phenotypes. Additionally, we should note that many investigations on the therapeutic strategies in disease mouse models clarify only a part of the relieved phenotypes, which could be because the researchers conducted only a part of the treatment evaluations or, more importantly, because the murine models have their own shortcomings for phenotypic assays. In fact, a high fraction of phenotypes in mice and human ID patients do not align well, for example, a subset of motor-functional deficits, neurodevelopmental and neurocognitive deficits involving intellectual impairments, behavioral disability, and sleep disorders.

Third, for potentially feasible management before clinical tests, the issues of the safety and tolerability of epigenetic drugs should be considered. In principle, the epigenetic reprogramming pathway modulating common epigenetic enzymes might generate a combination of opposite alterations depending on the consequences of the gain or loss of methylation in different genes. The low-specificity-caused general changes in the epigenetic pattern on non-targeted genes can lead to pleiotropic effects and chaotic disorders, which can be quite risky in humans. Therefore, epigenetic regulating agents and CRISPR-based epigenetic editing methods should be explored to collect detailed specificity information for humans and further narrow down potential off-target effects. While speculatively, risk–benefit assessments are necessary for the clinical use of novel epi-drugs. Another safety question is how to precisely control the restoration level of imprinted genes in the target tissue of patients because excessive gene dosages can introduce another risk factor for disruptions in the body; for example, UBE3A overexpression in the CNS can actually cause autism spectrum disorders (ASDs) [137, 138]. Hence, when the experimental epi-drugs move into the clinical study stage, they should be primarily utilized under careful supervision, and the sufficient therapeutic benefits of ameliorating or curing disease phenotypes with minimal adverse events should be monitored and evaluated precisely.

Fourth, the critical treatment window should be addressed when prescribed for ID patients with disturbed growth and development. Basically, epigenetics-based intervention would be an early-onset therapy because missing the critical treatment period may lead to less-than-expected therapeutic effects. To some extent, for CNS-related IDs, treatment during distinct neurodevelopmental windows can be a decisive factor in completely correcting pathological defects and acquiring sufficient rescue benefits. However, current studies are puzzling when using cell and animal models. On the one hand, although patient-derived iPSCs are reliable cell models reflecting the pathogenic conditions of early development, they cannot anticipate the situation in vivo. On the other hand, regarding disease-related mouse models, it is crucial to understand how the critical time window in humans compares to that in mice, which is not yet clear. Further investigation is needed to clarify the timing of therapeutic intervention in humans for each individual disease through clinical trials.

## Conclusions and future perspectives

In the past two decades, an improved understanding of both the fundamental mechanisms of genetic imprinting and genotype–phenotype correlations has enabled and driven unparalleled progress in developing novel therapeutics for human genetic IDs. The potential of innovative treatments to enhance patient benefit concentrates on overriding the limitations of current therapeutic strategies that are merely supportive and symptom-addressed. As focused upon in our review, these new and improved therapeutic approaches can be summarized in two primary directions: (1) exploiting symptomatic pharmacologic drugs targeting related pathophysiological pathways to treat specific symptom domains in genetic IDs, a large proportion of which have been developed in clinical trials and (2) exploring disease-modifying interventions of new techniques and therapeutic programs, such as gene replacement, molecular reinstatement of imprinted genes (through restoring the normal transcriptional activity of candidate genes or silencing the related inhibitory transcripts of imprinted genes), and epigenetics-based interventions. The latter therapeutic direction theoretically provides a more valid and promising treatment option for patients with genetic IDs. Specifically, it is tempting to imagine that the epi-based treatment approach could revolutionize the therapeutic strategy for genetic IDs and that, in the near future, the first application of epi-drugs might emerge as a supportive combination therapy to manage symptoms and limit disease progression for patients.

However, these emerging therapeutics are still at the discovery level, coping with various formidable obstacles. Going forward, on the one hand, more insights into the detailed molecular mechanism of genetic imprinting are still needed, providing the mechanistic basis and determining the relevance of potential therapeutic targets; on the other hand, more preclinical and clinical tests on discovered novel interventions are crucial for progress in this area. Undoubtedly, these new avenues of therapeutics open the door for treating genetic IDs and hold great promise for future adoption as accepted management strategies for patients.

## Data Availability

All the information is included in this manuscript.

## Abbreviations

AAV: Adeno-associated virus
ART: Assisted reproductive technology
AS: Angelman syndrome
ASDs: Autism spectrum disorders
ASOs: Antisense oligonucleotides
ATFs: Artificial transcription factors
BBB: Blood–brain barrier
BK: Calcium- and voltage-dependent big potassium
BWS: Beckwith–Wiedemann syndrome
CBD: Cannabidiol
CB1R: Cannabinoid type1 receptor
CDKN1C: Cyclin-dependent kinase inhibitor 1C
CFE: Caralluma fimbriata extract
Chr: Chromosome
CIM6P: Cation-independent mannose-6-phosphate
CNS: Central nervous system
CRISPR–Cas: Clustered regularly interspaced short palindromic repeats (CRISPR)–CRISPR-associated endonuclease (Cas)
CYP: Cyproheptadine
DBD: DNA-binding domain
dCas9: Dead Cas9
DCCR: Diazoxide choline controlled release
DIO3: Iodothyronine deiodinase 3
DLK1: Delta-like 1 homologue
DMD: Duchenne muscular dystrophy
DMRs: Differentially methylated regions
DPP4: Dipeptidyl peptidase-4
DSBs: Double-strand breaks
EEG: Electroencephalography
EHMT2/G9a: Euchromatic histone-lysine *N*-methyltransferase-2
GLP-1: Glucagon-like peptide 1
GNAS: Which encodes the G protein *α*-subunit Gs*α*
GOAT: Ghrelin *O*-acyltransferase
GRB10: Growth factor receptor-bound protein 10
GTL2: Gene-trap locus 2
hATTR: Hereditary transthyretin-mediated
HDR: Homologous-directed repair
HSPCs: Hematopoietic stem and progenitor cells
hUBE3A: Human UBE3A
HYMAI: Hydatidiform mole-associated and imprinted
H19: Which encodes an imprinted maternally expressed non-coding transcript
H3K9: Histone H3 lysine 9
H3K9me2: H3K9 di-methylation
H3K9me3: H3K9 tri-methylation
H19: Which encodes an imprinted maternally expressed non-coding transcript
ICR: Imprinting control region
ICSI: Intracytoplasmic sperm injection
IDs: Imprinting disorders
IGF2: Insulin-like growth factor 2
Igf2r: Insulin-like growth factor type 2 receptor
IG-DMR: Intergenic differentially methylated region
iPSCs: Induced pluripotent stem cells
IUGR: Intrauterine growth restriction
IVF: In vitro fertilization
KCNQ1: Potassium voltage-gated channel subfamily *Q* member 1
KCNQ1OT1: KCNQ1 opposite strand 1
KOS14: Kagami–Ogata syndrome
LCA: Leber congenital amaurosis
lncRNAs: Long non-coding RNAs
LOM: Loss of methylation
LV: Lentiviral vector
MAGEL2: MAGE family member *L*2
mat: Maternal
MC4R: Melanocortin-4 receptor
MEST: Mesoderm-specific transcript
MetAP2: Methionine aminopeptidase 2
miRNAs: MicroRNAs
MLIDs: Multi-locus imprinting disturbances
ncRNAs: Non-coding RNAs
NHEJ: Non-homologous end joining
NNZ-2591: Cyclo-l-glycyl-l-2-allylproline
NSI-189: NSI-189 phosphate
OMIM: Online Mendelian Inheritance in Man
pat: Paternal
PDE: Phosphodiesterase
PHP: Pseudohypoparathyroidism
PLAGL1: Pleomorphic adenoma gene‑like 1
PP2A: Protein phosphatase 2A
PTH: Parathyroid hormone
PWS: Prader–Willi syndrome
RobT: Robertsonian translocation
RTL1: Retrotransposon-like 1
SETDB1: SET domain bifurcated 1
sgRNAs: Single-guide RNAs
SMA: Spinal muscular atrophy
SNORD116: Small nucleolar RNA, *C*/*D* box 116 cluster
snoRNAs: Small nucleolar RNAs
SNRPN: Small nuclear ribonucleoprotein polypeptide *N*
SRS: Silver–Russell syndrome
SU: Sulfonylurea
SYS: Schaaf–Yang syndrome
TALEs: Transcription activator-like effectors
TET1: DNA plus dioxygenase 1
TNDM: Transient neonatal diabetes mellitus
TSS-DMR: Transcription start site differentially methylated region
TS14: Temple syndrome
UAG: Unacylated ghrelin
UBE3A: Ubiquitin protein ligase E3A
UBE3A-ATS: UBE3A antisense transcript
UPD: Uniparental disomy
ZNF274: Zinc finger protein 274
